# Estimation of glomerular filtration rate from serum creatinine and cystatin C in octogenarians and nonagenarians

**DOI:** 10.1186/1471-2369-14-265

**Published:** 2013-12-02

**Authors:** Marcelo B Lopes, Lara Q Araújo, Michelle T Passos, Sonia K Nishida, Gianna M Kirsztajn, Maysa S Cendoroglo, Ricardo C Sesso

**Affiliations:** 1 Nephrology, School of Medicine, Federal University of São Paulo, São Paulo, Brazil; 2 Geriatrics Divisions, Paulista School of Medicine, Federal University of São Paulo, Rua Botucatu 740, 04023-900, São Paulo, Brazil

**Keywords:** Chronic kidney disease, Creatinine, Cystatin C, Elderly, Glomerular filtration rate, Iohexol

## Abstract

**Background:**

Equations to estimate GFR have not been well validated in the elderly and may misclassify persons with chronic kidney disease (CKD). We measured GFR and compared the performance of the Modification of Diet in Renal Disease (MDRD), the Chronic Kidney Disease-Epidemiology Collaboration (CKD-Epi) and the Berlin Initiative Study (BIS) equations based on creatinine and/or cystatin C in octogenarians and nonagenarians.

**Methods:**

Using cross-sectional analysis we assessed 95 very elderly persons (mean 85 years) living in the community. GFR was measured by iohexol (mGFR) and compared with estimates using six equations: MDRD, CKD-Epi_creatinine, CKD-Epi_cystatin, CKD-Epi_creatinine-cystatin, BIS_creatinine and BIS_creatinine-cystatin.

**Results:**

Mean mGFR was 55 (range,19–86) ml/min/1.73 m^2^. Bias was smaller with the CKD-Epi_creatinine-cystatin and the CKD-Epi_creatinine equations (-4.0 and 1.7 ml/min/1.73 m^2^). Accuracy (percentage of estimates within 30% of mGFR) was greater with the CKD-Epi_creatinine-cystatin, BIS_creatinine-cystatin and BIS_creatinine equations (85%, 83% and 80%, respectively). Among the creatinine-based equations, the BIS_creatinine had the greatest accuracy at mGFR < 60 ml/min/1.73 m^2^ and the CKD-Epi_creatinine was superior at higher GFRs (79% and 90%, respectively). The CKD-Epi_creatinine-cystatin, BIS_creatinine-cystatin and CKD-Epi_cystatin equations yielded the greatest areas under the receiver operating characteristic curve at GFR threshold = 60 ml/min/1.73 m^2^ (0.88, 0.88 and 0.87, respectively). In participants classified based on the BIS_creatinine, CKD-Epi_cystatin, or BIS_creatinine-cystatin equations, the CKD-Epi_creatinine-cystatin equation tended to improve CKD classification (net reclassification index: 12.7%, p = 0.18; 6.7%, p = 0.38; and 15.9%; p = 0.08, respectively).

**Conclusions:**

GFR-estimating equations CKD-Epi_creatinine-cystatin and BIS_creatinine-cystatin showed better accuracy than other equations using creatinine or cystatin C alone in very elderly persons. The CKD-Epi_creatinine-cystatin equation appears to be advantageous in CKD classification. If cystatin C is not available, both the BIS_cr equation and the CKD-Epi_cr equation could be used, although at mGFR < 60 ml/min/1.73 m^2^, the BIS_cr equation seems to be the best alternative.

## Background

Chronic kidney disease (CKD) is a major public health problem and its prevalence is rising, particularly in the elderly, reflecting mainly the worldwide increased life expectancy of the population [1,2]. The measurement of kidney function remains challenging and CKD is imprecisely defined in older adults. Most previous studies in the elderly [3] have used creatinine-based equations to estimate GFR (eGFR), but these equations may be particularly limited due to non-GFR determinants of serum creatinine such as presence of chronic illnesses, decreased muscle mass and diet [4]. The relative imprecision of creatinine-based GFR estimates can potentially result in misclassification of individuals as having CKD, leading to unnecessary diagnostic and therapeutic interventions.

Cystatin C is considered to be a potential alternative to serum creatinine for estimating GFR [5,6]. Evidence suggests that cystatin C is less dependent upon muscle mass than creatinine and it is assumed that it should provide more accurate GFR estimates, particularly in populations with reduced muscle mass such as the elderly [5-7]. There has been limited evaluation of the GFR estimating equations in the elderly [8]. Recently the Berlin Initiative Study (BIS) developed two novel equations to estimate kidney function in older people [9], but they have not been validated in other data sets.

The aims of this study were to measure GFR, to compare the performance of some frequently used or recently described GFR estimating equations based on creatinine and cystatin C, alone or combined, and test their usefulness in the classification of CKD in individuals 80 years of age or older.

## Methods

### Study population

This is a cross sectional study of community-dwelling independent elderly aged 80 years or older recruited in the southern region of the city of São Paulo, Brazil. The elderly were identified through regional surveys among those individuals living in the neighborhood of the Federal University of São Paulo campus from November 2010 through December 2011. During this period, 294 elderly were identified and after interview and physical examination 200 were enrolled in a longitudinal epidemiologic study at the Department of Geriatrics. All clinically stable individuals and independent in activities of daily living were acceptable to participate in the study. The exclusion criteria were: to be institutionalized, unable or unwilling to give consent, to have an acute infectious disease, a moderate or severe cognitive impairment, heart failure (NYHA class III or IV), known cirrhosis, previously received dialysis, unstable chronic pulmonary disease, previous immunosuppressive therapy within 6 mo., previous chemotherapy for cancer, known HIV infection, and previously reported allergic reaction to iodine. From August 2011 to May 2012, of the 294 initial individuals, 94 were excluded after checking the selection criteria. The purpose and the procedures for this study were explained by telephone to the remaining 200 and, of these, 97 returned to the clinics for the renal function studies scheduled within a week of the date of interview. There were no sociodemographic or clinical characteristics significantly different between the participants (n = 97) and the remaining 103 individuals enrolled in the epidemiologic Geriatric study that did not undergo renal function tests. The study protocol was approved by the Research Ethics Committee of the Federal University of São Paulo and all participants gave their written informed consent.

### Analytical methods

#### *Determination of serum creatinine*

After the blood sample was collected from each participant, the material was immediately processed and analyzed in the same day and always in the same laboratory at the university Hospital do Rim. Serum creatinine was measured using a modified kinetic Jaffé colorimetric method on an autoanalyzer (Beckman Coulter AU 400, CA, USA), which was calibrated to isotope dilution mass spectrometry (IDMS) using a standard reference material (914a) traceable to the National Institutes of Standards and Technology (NIST). Total analytical variation (CV%) of the colorimetric method was 1.4-3.0%. The intra-assay coefficient variation (CV) for creatinine was 1.9%.

#### *Determination of plasma cystatin C*

Plasma cystatin C levels were determined by an automated particle-enhanced immunoturbidimetric method [6] using a Beckman AU 400 analyzer (Beckman Coulter, Inc. CA, USA), reagents (code Nos. LX002, s2361, X0973, X0974) obtained from DakoCytomation (Glostrup, Denmark) and following the procedures recommended by the reagent producer. The intra-assay CV was 4.7% and the inter-assay CV was 5.2% at a cystatin C level of 1.0 mg/L. All samples were frozen at -70°C and analyzed within 7 days in May 2012.

#### *Determination of iohexol clearance *[10]

Five mL of iohexol (Omnipaque 300 mg iodine/mL, GE Healthcare) were administered intravenously in an antecubital vein and blood samples were obtained at 2, 3, 4 and 5 hours after infusion. Iohexol clearance (referred to as ‘measured GFR’ (mGFR)) was calculated based on the measured decline in plasma concentrations of iohexol, using the slope intercept method (one-compartment model) [11]. Plasma iohexol concentrations were determined by a capillary electrophoresis method [12] in an automated instrument (5010 Beckman Instruments, Palo Alto, CA). The inter-assay CV was between 5.7% and 7.4%, and the intra-assay CV were between 3.4 and 4.0%. Measured GFR values were adjusted to 1.73 m^2^ body surface area [13].

#### *GFR estimating equations used (Table *1)

**Table 1:** GFR estimating equations used in the study

1.	The Modification Diet in Renal Disease (MDRD) equation [14]: 175 × Scr^‒ 1.154^ × age^‒ 0.203^ × 0.742(if female) × 1.212(if black); where Scr is serum creatinine, mg/dl; age in years.
2.	The Chronic Kidney Disease Epidemiology creatinine (CKD-Epi_cr) equation [15]: 141 × min(Scr/κ, 1)^a^ × max(Scr/κ, 1)^‒ 1.209^ × 0.993^age^ × 1.018(if female) × 1.159(if black); where Scr is serum creatinine, k is 0.7 for females and 0.9 for males, α is -0.329 for females and -0.411 for males, min is the minimum of Scr/ k or 1, and max is the maximum of Scr/k or 1.
3.	The Chronic Kidney Disease Epidemiology cystatin C (CKD-Epi_cys) equation [16]: 133 × min(Scys/0.8, 1)^‒ 0.499^ × max(Scys/0.8, 1)^‒ 1.328^ × 0.996^age^(×0.932 if female); where Scys is serum cystatin C, min indicates the minimum of Scr/κ or 1, and max indicates the maximum of Scys/κ or 1.
4.	The Chronic Kidney Disease Epidemiology creatinine-cystatin C (CKD-Epi_cr-cys) equation [16]: 135 × min(Scr/κ, 1)^α^ × max(Scr/κ, 1)^‒ 0.601^ × min(Scys/0.8, 1)^‒ 0.375^ × max(Scys/0.8, 1)^‒ 0.711^ × 0.995^age^(×0.969 if female)(×1.08 if black); where Scr is serum creatinine, Scys is serum cystatin C, κ is 0.7 for females and 0.9 for males, α is -0.248 for females and -0.207 for males, min indicates the minimum of Scr/κ or 1, and max indicates the maximum of Scr/κ or 1.
5.	The Berlin Initiative Study creatinine (BIS_cr) equation [9]: 3736 × creatinine^‒ 0.87^ × age^‒ 0.95^ × 0.82(if female).
6.	The Berlin Initiative Study creatinine-cystatin C (BIS_cr-cys) equation [9]: 767 × cystatin C^‒ 0.61^ × creatinine^‒ 0.40^ × age^‒ 0.57^ × 0.87(if female).

1. The Modification Diet in Renal Disease (MDRD) equation [14].

2. The Chronic Kidney Disease Epidemiology creatinine (CKD-Epi_cr) equation [15].

3. The Chronic Kidney Disease Epidemiology cystatin C (CKD-Epi_cys) equation [16].

4. The Chronic Kidney Disease Epidemiology creatinine-cystatin C (CKD-Epi_cr-cys) equation [16].

5. The Berlin Initiative Study creatinine (BIS_cr) equation [9].

6. The Berlin Initiative Study creatinine-cystatin C (BIS_cr-cys) equation [9].

### Statistical analysis

Values are expressed as mean ± SD or median and interquartile range. Bias, precision and accuracy were measured to determine the performance of each equation [17]. Bias was assessed as the mean of the difference between estimated GFR and measured GFR. The width of the SD of the mean difference is an estimation of precision. Accuracy was assessed as the percentage of results that did not deviate more than 30% from the measured GFR and also as the combined root mean square error (CRMSE). These analyses were performed in the whole group and in subgroups according to mGFR cutoff of 60 ml/min/1.73 m^2^. The significance of the differences among equations was determined by the paired Student t test, and McNemar’s test for differences in proportions.

The diagnostic accuracy of eGFR equations was evaluated using receiver operating characteristics (ROC) plots. The GFR determined with iohexol clearance was used as the gold standard and the cutoff value was set at 60 ml/min/1.73 m^2^ for CKD [17]. Analyses were also performed for the cutoff value of 45 ml/min/1.73 m^2 ^[18].

We assessed the use of the equations with best performances for the classification of CKD by means of the net reclassification index (NRI) statistic. We compared the proportion of participants who were reclassified as having a measured GFR that was less than 60 (or 45) ml/min/1.73 m^2^ on the basis of one equation versus the other. Analyses were performed with the use of SPSS version 17 (SPSS, Chicago, IL, USA) and MatLab version 5 (The MathWorks Inc. Natick, MA, USA), software.

## Results

Of the 97 elderly who underwent the renal function studies, two were excluded due to incorrect technical procedures for the iohexol determination, resulting in a sample of 95 participants for the analyses. The characteristics of the participants are shown in Table 2. The average age of the sample was 85 years, ranging from 80 to 97 years, 70% were women, 93% Caucasians.

**Table 2:** Characteristics of the study participants, parameters of renal function, measured GFR with iohexol and estimated GFR with creatinine- and cystatin C-based equations

**Parameter**	**N = 95**
Age, yr	85.3 ± 4.3 (80–97)
Age, yr	
80–89	78 (82)
90–100	17 (18)
Female sex	67 (70)
Black race	7 (7)
Diabetes	22 (23)
Weight, kg	64.3 ± 13.7
Height, cm	155 ± 10
BMI, kg/m^2^	26.4 ± 4.4
<20	5 (5)
20–24	27 (29)
25–30	44 (46)
>30	19 (20)
Body surface area, m^2^	1.62 ± 0.21
Creatinine, mg/dL	1.08 ± 0.37
Cystatin C, ml/L	1.44 ± 0.45
Measured GFR, ml/min/1.73 m^2^	55 ± 15 (44–65)
<15	0
15–29	4 (4)
30–44	22 (23)
45–59	30 (32)
60–89	39 (41)
MDRD, ml/min/1.73 m^2^	60 ± 19 (45–74)
<60	50 (53)
CKD-Epi creatinine, ml/min/1.73 m^2^	57 ± 18 (44–72)
<60	51 (54)
BIS creatinine, ml/min/1.73 m^2^	48 ± 12 (40–57)
<60	79 (83)
CKD-Epi cystatin C, ml/min/1.73 m^2^	48 ± 19 (35–57)
<60	75 (79)
CKD-Epi creatinine-cystatin C,	51 ± 17 (39–62)
ml/min/1.73 m^2^	
<60	69 (73)
BIS creatinine-cystatin C,	47 ± 12 (38–54)
ml/min/1.73 m^2^	
<60	82 (86)

Values expressed as mean ± SD (interquartile range, except for age where range is minimum-maximum) or N (%). GFR: glomerular filtration rate. BMI: body mass index; weight in kg divided by the square of the height in meters.

**Figure 1:**
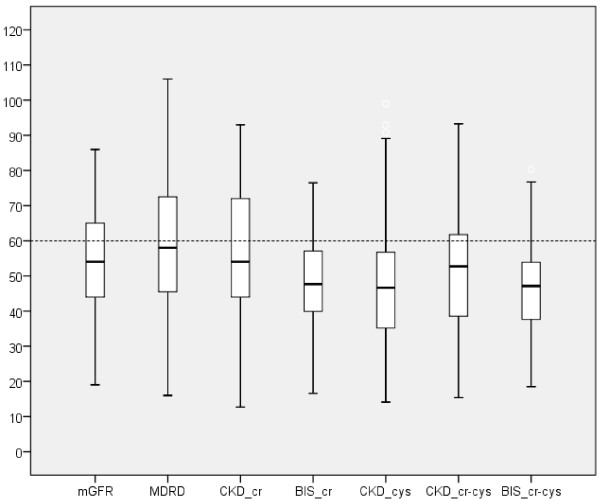
**Boxplot of measured and estimated GFR according to several equations.** Boxes indicate medians (line inside box), quartiles (upper and lower margins of the box). Vertical lines are 95% confidence intervals. The dotted line represents the GFR cutoff of 60 ml/min/1.73 m^2^. Abbreviations: mGFR: measured GFR with iohexol, MDRD: Modification Diet in Renal Disease equation, CKD_cr: Chronic Kidney Disease Epidemiology creatinine equation, BIS_cr: Berlin Initiative Study creatinine equation, CKD_cys: Chronic Kidney Disease Epidemiology cystatin C equation, CKD_cr-cys: Chronic Kidney Disease Epidemiology creatinine-cystatin C equation, BIS_cr-cys: Berlin Initiative Study creatinine-cystatin C equation.

Mean(±SD) iohexol clearance (mGFR) was 55 ± 15 ml/min/1.73 m^2^. Fifty-nine percent had mGFR <60 ml/min/1.73 m^2^ (Table 2). A higher percentage of participants had eGFR < 60 ml/min/1.73 m^2^ with the BIS_cr-cys equation and a lower percentage with the MDRD equation. Performance of the equations is summarized in Table 2, and in the boxplots shown in Figure 1. In the overall group, the MDRD and the CKD-Epi_cr equations overestimated and all the others underestimated GFR. The least biased equation was the CKD-Epi_cr, whereas the BIS_cr-cys and the CKD-Epi_cys were the most biased. The most precise equations were the BIS_cr-cys, the BIS_cr and the CKD-EPI_cr-cys. In the analysis of accuracy (CRMSE) the equations with best performances were the CKD-Epi_cr-cys, the BIS_cr and the CKD-Epi_cr. The most accurate equations (greater percentage of estimates within 30% of mGFR) were the CKD_cr-cys and the BIS_cr-cys (85% and 83%, respectively); and significantly inferior results were observed with the CKD-Epi_cys, the MDRD and the CKD-Epi_cr equations.

Similar trends were in general observed when these analyses were repeated according to mGFR < or ≥60 ml/min/1.73 m^2^ (Table 3). However, the BIS equations and the CKD-Epi_cr-cys had less bias, and the MDRD was more biased when mGFR was <60 ml/min/1.73 m^2^. The opposite was observed with mGFR ≥60 ml/min/1.73 m^2^. The best performances combining bias and precision (CRMSE) were obtained with both BIS equations and the CKD_cr-cys when GFR was <60 ml/min/1.73 m^2^; and with the CKD-Epi_cr-cys and the CKD-Epi_cr in the higher GFR subgroup. As for the accuracy within 30% the best performances were obtained with the BIS_cr-cys and the CKD-Epi_cr-cys equations at lower GFR levels; and the CKD-Epi_cr-cys and the CKD-Epi_cr at when GFR was ≥60 ml/min/1.73 m^2^ (92% and 90%, respectively). The BIS_cr equation had a more stable performance in the two investigated GFR subgroups than the other creatinine-based equations and showed better accuracy at decreased mGFR levels.

**Table 3:** **Bias, precision and accuracy of the estimating equations in the whole group and according to the glomerular filtration rate cut-off value of 60 ml/min/1.73 m**^**2 **^**determined by iohexol clearance**

**Variable/equations**	**Overall**	**mGFR <60 ml/min/1.73 m**^**2**^	**mGFR ≥60 ml/min/1.73 m**^**2**^
	**(N = 95)**	**(N = 56)**	**(N = 39)**
Bias			
MDRD	4.6 (1.7 to 7.5)*	5.9 (2.2 to 9.7)*	2.7 (-2.1 to 7.5)
CKD-Epi creatinine	1.7 (-1.0 to 4.4)	3.6 (1.8 to -0.1)	-1.0 (-5.1 to 3.1)
BIS creatinine	-6.6 (-8.9 to -4.3)*	-1.9 (-4.6 to 0.9)	-13.4 (-16.4 to -10.4)*
CKD-Epi cystatin C	-7.4 (-10.3 to -4.4)*	-6.3 (-9.9 to -2.7)*	-8.9 (-14.1 to -3.7)*
CKD-Epi creatinine-cystatin C	-4.0 (-6.4 to -1.6)*	-2.7 (-5.9 to 0.5)	-5.9 (-9.6 to -2.3)*
BIS creatinine-cystatin C	-8.3 (-10.5 to -6.0)*	-4.3 (-7.0 to -1.6)*	-13.9 (-17.1 to -10.7)*
Precision			
MDRD	14.4 (12.6 to 16.1)	14.1 (11.5 to 16.5)	14.8 (12.0 to 16.8)
CKD-Epi creatinine	13.3 (11.7 to 14.9)	13.6 (11.2 to 15.7)	12.7 (10.3 to 14.4)
BIS creatinine	11.4 (10.0 to 12.6)	10.4 (8.6 to 12.0)	9.3 (7.8 to 10.4)
CKD-Epi cystatin C	14.5 (11.6 to 17.7)	13.4 (9.0 to 18.7)	16.1 (12.1 to 18.8)
CKD-Epi creatinine-cystatin C	11.7 (9.7 to 14.1)	12.0 (9.0 to 15.4)	11.3 (7.9 to 13.4)
BIS creatinine-cystatin C	11.0 (9.3 to 12.9)	10.1 (7.7 to 12.9)	9.9 (7.4 to 11.8)
CRMSE			
MDRD	15.1 (13.9 to 16.3)	15.3 (13.5 to 17.2)	15.0 (13.5 to 16.5)
CKD-Epi creatinine	13.5 (12.5 to 14.5)	14.0 (12.5 to 15.5)	12.7 (11.4 to 14.0)
BIS creatinine	13.2 (12.1 to 14.3)	10.6 (9.5 to 11.7)	16.3 (14.2 to 18.4)
CKD-Epi cystatin C	16.3 (15.0 to 17.6)	14.8 (13.0 to 16.6)	18.4 (16.4 to 20.4)
CKD-Epi creatinine-cystatin C	12.4 (11.4 to 13.4)	12.3 (10.8 to 13.7)	12.7 (11.3 to 14.1)
BIS creatinine-cystatin C	13.8 (12.7 to 14.9)	11.2 (9.9 to 12.5)	17.1 (15.1 to 19.1)
Accuracy within 30%,%			
MDRD	70.5 (60.7 to 78.7)^ad^	64.3 (51.2 to 75.5)^bcf^	79.5 (64.5 to 89.2)
CKD-Epi creatinine	74.7 (65.2 to 82.3)^b^	64.3 (51.2 to 75.5)^bce^	89.7 (76.4 to 95.9)
BIS creatinine	80.0 (70.9 to 86.8)	78.6 (66.2 to 87.3)	82.1 (67.3 to 91.0)
CKD-Epi cystatin C	65.3 (55.3 to 74.1)^acf^	60.7 (47.6 to 72.4)^bc^	71.8 (56.2 to 83.4)^b^
CKD-Epi creatinine-cystatin C	85.3 (77.0 to 91.3)	80.4 (68.2 to 88.7)	92.3 (79.7 to 97.3)
BIS creatinine-cystatin C	83.2 (74.6 to 89.7)	85.7 (74.3 to 92.6)	79.5 (64.5 to 89.2)

Values are expressed in ml/min/1.73 m^2^, except for accuracy where values are % of subjects. In parenthesis are 95% confidence intervals.

mGFR: glomerular filtration rate measured by iohexol clearance.

CRMSE: combined root mean square error = the square root of [(mean difference between estimated and measured GFR)^2^ + (SD of the difference)^2^].

*p < 0.01 compared with mGFR.

^a^p < 0.01, ^b^p < 0.05 compared with the CKD-Epi creatinine-cystatin C equation.

^c^p < 0.01, ^d^p < 0.05 compared with the BIS creatinine-cystatin C equation.

^e^p < 0.01, ^f^p < 0.05 compared with the BIS creatinine equation.

The performance of the equations in relation to the classification of the thresholds of mGFR was assessed by the area under the ROC curve (Table 4). The equations with the best sensitivity (correct identification of individuals with mGFR <60 ml/min/1.73 m^2^) were the BIS_cr-cys, BIS_cr, and the CKD-Epi_cys; and the best specificity was achieved with the MDRD and the CKD-Epi_cr equations. The greatest area under the ROC curve was observed with the CKD-Epi_cr-cys and the BIS_cr-cys equations (0.88) followed by the CKD-Epi_cys (0.87) equation. Although the area under the curve of the CKD-Epi_cr-cys was greater compared with the MDRD and the CKD-Epi_cr equations, the differences were not significant (p = 0.06 and p = 0.09, respectively). Considering the cutoff point of 45 ml/min/1.73 m^2^ the greatest area under the curve was again obtained with the CKD-Epi_cr-cys (0.81) equation.

**Table 4:** **Sensitivity, specificity and area under the ROC curves at mGFR cut-off values of 60 ml/min/1.73 m**^**2 **^**and 45 ml/min/1.73 m**^**2 **^**for estimating equations**

**Equation**	**Sensitivity**	**Specificity**	**Area under the curve**
	**%**	**%**	
MDRD			
mGFR < 60 ml/min/1.73 m^2^	75.0	79.5	0.832 ± 0.042*
mGFR < 45 ml/min/1.73 m^2^	38.5	82.6	0.763 ± 0.049
CKD-Epi creatinine			
mGFR < 60 ml/min/1.73 m^2^	76.8	79.5	0.836 ± 0.042^†^
mGFR < 45 ml/min/1.73 m^2^	46.2	81.2	0.756 ± 0.050^¶^
BIS creatinine			
mGFR < 60 ml/min/1.73 m^2^	96.4	35.9	0.841 ± 0.042
mGFR < 45 ml/min/1.73 m^2^	65.4	66.7	0.755 ± 0.050^¶^
CKD-Epi cystatin C			
mGFR < 60 ml/min/1.73 m^2^	94.6	43.6	0.873 ± 0.035
mGFR < 45 ml/min/1.73 m^2^	73.1	65.2	0.760 ± 0.057^‡^
CKD-Epi creatinine-cystatin C			
mGFR < 60 ml/min/1.73 m^2^	91.1	53.8	0.884 ± 0.034
mGFR < 45 ml/min/1.73 m^2^	76.9	71.0	0.813 ± 0.049
BIS creatinine-cystatin C			
mGFR < 60 ml/min/1.73 m^2^	98.2	30.8	0.876 ± 0.035
mGFR < 45 ml/min/1.73 m^2^	76.9	68.1	0.772 ± 0.052^§^

Values for the area under the curve are ± SE.

p-values for the comparisons of the area under the curve for the equations at the cut-off value of 60 ml/min/1.73 m^2^:

*p < 0.06; ^†^p = 0.09 compared with CKD-Epi creatinine-cystatin C.

p-values for the comparisons of the area under the curve for the equations at the cut-off value of 45 ml/min/1.73 m^2^: ^§^p = 0.05; ^‡^p = 0.06, ^¶^p = 0.08 compared with CKD-Epi creatinine-cystatin C.

We then assessed the use of the CKD-Epi_cr-cys combined equation for the reclassification of participants as having a measured GFR < or ≥60 ml/min/1.73 m^2^ on the basis of the other equations (Additional file 1: Table S1). In general the CKD-Epi_cr-cys equation more often correctly reclassified those with mGFR ≥60 ml/min/1.73 m^2^, but the net reclassification index was not statistically significant with the CKD-Epi_cr-cys equation as compared with the CKD-Epi_cys (NRI = 6.7, p = 0.38), BIS_cr (NRI = 12.7, p = 0.18) or the BIS_cr-cys (NRI = 15.9, p = 0.08) equations. Compared with the CKD-Epi_cr, reclassification with the combined CKD-Epi_cr-cys equation was not favorable and was more often incorrect in those with a mGFR ≥60 ml/min/1.73 m^2^ (NRI = -11.4, p = 0.30). In a similar analyses for reclassification based on a mGFR threshold of 45 ml/min/1.73 m^2^ the CKD-Epi_cr-cys equation tended to improve classification compared with all other equations; the NRI was statistically significant compared with the BIS_cr (NRI = 15.8, p = 0.04), marginally significant compared with the CKD-EPI_cr (NRI = 20.6, p = 0.08), and not significant versus the CKD-Epi_cys (NRI = 9.7, p = 0.20), and the BIS_cr-cys equation (NRI = 2.9, p = 0.16) (Additional file 2: Table S2).

## Discussion

Accurate assessment of GFR is essential for the interpretation of clinical and laboratory abnormalities that may indicate CKD, for drug dosing, for diagnosis, management of CKD as well as establishing prognosis. The definition of CKD in the elderly has been a matter of debate. It has been questioned the appropriateness of the arbitrary cutoff value of eGFR < 60 ml/min/1.73 m^2^ for the definition of CKD, without some adjustment for the normal gender-specific decline in eGFR with aging [19]. Although a reduction in GFR to <60 ml/min/1.73 m^2^ has been associated with worse outcomes, a lower cutoff of 45 ml/min/1.73 m^2^ has been proposed for the elderly [18].

In the present study conducted in a sample of community-dwelling octogenarians and nonagenarians, we found a mean mGFR of 55 ml/min/1.73 m^2^, 59% of whom with values <60 ml/min/1.73 m^2^. The percentage of patients with eGFR <60 ml/min/1.73 m^2^ varied widely from 53% to 86% depending on the estimating equation used, suggesting caution in the interpretation of the estimates.

Most studies estimating GFR in the elderly have used equations based on serum creatinine; nevertheless, it is well known that the decreased muscle mass frequently occurring in the elderly affects creatinine generation and influences estimates [3,4]. Inflammation, malnutrition and loss of muscle bulk associated with chronic diseases can further accentuate muscle metabolic abnormalities and influence the value of creatinine-based equations [3,4].

Among the creatinine based equations assessed, the MDRD equation overestimates mGFR and its use will decrease the diagnosis of CKD. It tended to be less accurate than the CKD-Epi_cr and the BIS_cr equations as previously reported [8,9,15,20], especially at eGFR >60 ml/min/1.73 m^2^ compared with the CKD-Epi_cr and at lower GFRs compared with the BIS_cr. It should be noted that the MDRD study population is considerably younger and does not include persons older than 70 years, but the CKD-Epi Collaboration study does include older adults; and the CKD-Epi_cr equation was developed in a cohort with better kidney function (68 ml/min/1.73 m^2^). Some studies have reported that both the MDRD and, to a lesser extent, the CKD-Epi_cr equations overestimate GFR in individuals older than 70 years with CKD [8,20,21]. The BIS_cr equation underestimated mGFR and more often than the others incorrectly classified participants as having CKD. Overall the accuracy of the BIS_cr was slightly superior to the CKD-Epi_cr equation, but the difference was not as favorable to the former as described in the BIS original validation cohort [9]. Our results suggest that the BIS_cr equation outperforms the MDRD and the CKD-Epi_cr equations in terms of precision. Notably, the BIS_cr equation had a superior performance compared with the other two at decreased GFR levels, mainly due to increased precision; but yielded substantial underestimation in participants with mGFR ≥60 ml/min/1.73 m^2^. The marker used for measuring clearance and the methodology of measuring creatinine were the same in our study and in the BIS and do not seem to explain these differences. One could speculate that residual differences in creatinine standardization (eventually more influential at low creatinine levels) or differences in ethnicity, since the BIS participants were white Europeans, while our population is less homogeneous in terms of ethnicity, might play a role in the distinct findings. In fact, ethnicity influences creatinine-based GFR estimating equations. Flamant et al. [22] have suggested that eGFR (CKD-Epi_cr equation) with the African American race-ethnicity correction factor overestimates GFR in African Europeans. Recently Koppe et al. [23] reported that the BIS_cr equation in comparison the MDRD and the CKD-Epi_cr was the most reliable to assess renal function in elderly patients, especially in those with CKD stages 1–3; however, no superior results were observed in those aged >80 years. The participants in that study had several differences compared with ours: there was a smaller number of very old individuals, a higher proportion of men, all subjects were white and had suspected or established renal disease.

Serum cystatin C appears to be less dependent upon muscle mass, less sensitive to metabolic and extra-renal factors than creatinine in the elderly and equations based on this marker seemed to be promising [24]. In contrast to early reports, there are non-GFR determinants of cystatin C serum levels [5]. The advantage of the recently described cystatin C-based equations over the creatinine-based equations is that it is less subject to the effects of age, sex and race [15,25]. Bevc et al. reported that in adult patients aged >65 years, a simple cystatin C formula (100/serum cystatin C) was reliable and comparable to creatinine-based formulas [26]. We observed that GFR estimates based on equations that use cystatin C as the sole filtration marker have not been more accurate than creatinine based estimates, as also reported in younger adult and elderly populations [8,16,25]. In fact, our data show that the CKD-Epi_cys performance tended to be inferior to the CKD-EPI_cr and this trend was more evident when compared with the BIS_cr equation. Equivalent performance of the CKD-Epi_cys and CKD-Epi_cr equations [8], and slightly better accuracy of the BIS_cr compared with the CKD-Epi_cys in the elderly have been reported [9]. This suggests that unmeasured and largely unknown non-GFR determinants of cystatin C serum levels are similar in magnitude to those of creatinine.

The combined creatinine-cystatin C equations (CKD-Epi_cr-cys and BIS_cr-cys) had the greatest accuracy (values within 30%), although both (more markedly the BIS_cr-cys) underestimated GFR. As for the classification of CKD, the combined equations performed better than the others, followed by the CKD-Epi_cys. The ROC analyses revealed that the combined equations and the cystatin alone equation had the greatest areas under the curve at GFR =60 ml/min/1.73 m^2^ threshold. There was no significant difference in CKD classification comparing the equations using creatinine alone (CKD-Epi_cr and BIS_cr). The CKD-Epi_cr-cys combined equation tended to improve the reclassification of patients as having CKD compared with most of the others.

To explain the tendency of best performance with the equations that combine creatinine and cystatin C it has been proposed that errors due to the non-GFR determinants of creatinine and cystatin C are independent and smaller in an equation that uses both markers than in an equation that uses only one marker. Possible reasons for the continued imprecision are the residual contribution of non-GFR determinants of each marker, as well as physiologic variation in GFR and error in measurement of GFR.

Our study suggests that cystatin C should not replace creatinine in clinical practice; however, the combination of creatinine and cystatin C appears to provide more accurate GFR estimates in the very elderly. In adults, Inker et al. [16] showed that the combined creatinine-cystatin C equation could be used as a confirmatory test for the diagnosis of CKD. Kilbride et al. [8] have shown that the CKD-Epi equations (based on creatinine, cystatin C or both) have equivalent and satisfactory performance in older people; and worked as well as compared with younger populations. In fact, the accuracy that we obtained with the CKD-Epi_cr-cys equation (85%) was identical to that reported by those authors in individuals older than 80 years [8]. Schaeffner et al. [9] have recently suggested that the BIS_cr-cys combined equation should be used to estimate GFR in the elderly. However, their data need to be validated in external validation samples. Typically, an equation performs best in the data set from which it was derived. Our findings did not confirm the overall superiority of the BIS equations performance in relation to the CKD-Epi equations. It is possible that some characteristics of our study sample, the greater participation of women, the greater ethnic miscegenation, the higher mean age, the lower mean body surface area, the lower mean mGFR and the smaller sample size may have influenced these discrepant findings. In addition, the comparisons reported in the BIS study [9] were not with the most recently developed CKD-Epi_cys and CKD-Epi_cr-cys equations [16] as we did.

The strengths of this study are the evaluation of mGFR using a precise method in a sample of very elderly persons, and the comparison and validation of several recently proposed equations to estimate GFR based on creatinine and cystatin C. None of the previous studies have specifically assessed the comparison of these new equations in this age group. The limitations of the study are: the relatively small sample of octogenarians and nonagenarians, owing to the difficulties in recruiting individuals in this age group for a lengthy procedure such as the GFR determination, resulted in a lower power to detect statistically significant differences; the number of participants and the recruitment methods used restrict the representativeness of the population as a whole; errors in the measured GFR may account for some imprecision and limiting interpretation of the analyses. The cystatin C assay was not calibrated against the international certified reference material ERM-DA471/IFCC prior the use in GFR estimating equations; this lack of standardization may have caused imprecision in the estimates. Therefore, these results should be interpreted with caution. Although the most reliable method to analyze plasma iohexol is with high performance liquid chromatography, previous studies have shown that measurements by capillary electrophoresis had an excellent correlation with those by high performance liquid chromatography [12]. For reasons of feasibility and precision in an elderly cohort with a mean age of 85.3 years, we measured only plasma clearance and not urine clearance. Early studies suggest that tubular secretion of iohexol is negligible [27] and there is no convincing evidence that iohexol clearance is different than other reference GFR procedures compared to urinary inulin clearance [28].

## Conclusions

The combined CKD-Epi_cr-cys and BIS_cr-cys equations, although underestimating GFR, showed better accuracy than other equations using creatinine or cystatin C alone in very elderly persons. The CKD-Epi_cr-cys equation appears to be advantageous in CKD classification. If cystatin C is not available, both the BIS_cr equation and the CKD-Epi_cr equation could be used, although at mGFR < 60 ml/min/1.73 m^2^, the BIS_cr equation seems to be the best alternative.

## Abbreviations

CKD: Chronic kidney disease; GFR: Glomerular filtration rate; mGFR: Measured glomerular filtration rate; eGFR: Estimated glomerular filtration rate; MDRD: The modification diet in renal disease; CKD-Epi_cr: The Chronic kidney disease epidemiology creatinine; CKD-Epi_cys: The chronic kidney disease epidemiology cystatin C; CKD-Epi_cr-cys: The chronic kidney disease epidemiology creatinine-cystatin C; BIS_cr: The berlin initiative study creatinine; BIS_cr-cys: The berlin initiative study creatinine-cystatin C.

## Competing interests

The authors declare that they have no competing interest.

## Authors’ contributions

MBL participated in the conception, design of the study, data collection, data analysis, data interpretation, and approved the manuscript. LQA participated in the conception, design of the study, data collection, data interpretation, and approved the manuscript. MTP participated in the data analysis, laboratory parameters analysis, data interpretation, and approved the manuscript. SKN participated in the data analysis, laboratory parameters analysis, data interpretation, and approved the manuscript. GMK participated in the conception of the study, data analysis, data interpretation, provided important intellectual content, critically revised the manuscript, and approved the manuscript. MSC participated in the conception, design of the study, data collection, data interpretation, provided important intellectual content, critically revised the manuscript, and approved the manuscript. RCS participated in the conception, design of the study, data analysis, data interpretation, provided important intellectual content, and has written the manuscript. All authors read and approved the final manuscript.

## Pre-publication history

The pre-publication history for this paper can be accessed here:

http://www.biomedcentral.com/1471-2369/14/265/prepub

## Supplementary Material

Additional file 1: Table S1 Reclassification of the participants with the use of the CKD-Epi creatinine-cystatin C equation versus: the CKD-Epi creatinine, the CKD-Epi cystatin C, the BIS creatinine or the BIS creatinine-cystatin C for estimated GFR, according to the cut-off value of mGFR = 60 ml/min/1.73 m^2^. Click here for file

Additional file 2: Table S2 Reclassification of the participants with the use of the CKD-Epi creatinine-cystatin C equation versus: the CKD-Epi creatinine, the CKD-Epi cystatin C, the BIS creatinine or the BIS creatinine-cystatin C for estimated GFR, according to the cut-off value of mGFR = 45 ml/min/1.73 m^2^. Click here for file
