# Supplementary material for: Estimation of glomerular filtration rate from serum creatinine and cystatin C in octogenarians and nonagenarians
Source: BMC Nephrol. 2013 Dec 2;14:265. doi: 10.1186/1471-2369-14-265 (PMC4219437; doi:10.1186/1471-2369-14-265)
Supplement: Additional file 1: Table S1 — Reclassification of the participants with the use of the CKD-Epi creatinine-cystatin C equation versus: the CKD-Epi creatinine, the CKD-Epi cystatin C, the BIS creatinine or the BIS creatinine-cystatin C for estimated GFR, according to the cut-off value of mGFR = 60 ml/min/1.73 m2. [file 1471-2369-14-265-S1.doc]

Additional File 1. Table S1. Reclassification of the participants with the use of the CKD-Epi creatinine-cystatin C equation versus: the CKD-Epi creatinine, the CKD-Epi cystatin C, the BIS creatinine or the BIS creatinine-cystatin C for estimated GFR, according to the cut-off value of mGFR= 60 ml/min/1.73m^2^.

| Total group  N=95 | | Subgroup with mGFR <60 ml/min/1.73m^2^  N=56 | | | Subgroup with mGFR ≥60 ml/min/1.73m^2^  N=39 | | | NRI |
| --- | --- | --- | --- | --- | --- | --- | --- | --- |
| eGFR,  ml/min/1.73m^2^ | N (%)  Reclassified | Correctly Reclassified  N (%) | Incorrectly Reclassified  N (%) | Difference  (%) | Correctly Reclassified  N (%) | Incorrectly Reclassified  N (%) | Difference  (%) | (%) |
| CKD-Epi_cr | 22 (23.2) | ^a^  8 (14.3) | ^b^  0 (0) | 14.3 | ^c^  2 (5.1) | ^d^  12 (30.8) | -25.7 | -11.4^e^ |
| CKD-Epi_cys | 10 (10.5) | ^f^  0 (0) | ^g^  2 (3.6) | -3.6 | ^h^  6 (15.4) | ^i^  2 (5.1) | 10.3 | 6.7^j^ |
| BIS_cr | 16 (16.8) | ^k^  1 (1.8) | ^l^  4 (7.1) | -5.3 | ^m^  9 (23.1) | ^n^  2 (5.1) | 18.0 | 12.7^o^ |
| BIS_cr-cys | 15 (15.8) | ^p^  0 (0) | ^q^  4 (7.1) | -7.1 | ^r^  10 (25.6) | ^s^  1 (2.6) | 23.0 | 15.9^t^ |

eGFR= estimated GFR, CKD-Epi_cr= estimated GFR from CKD-Epi creatinine equation; CKD-Epi_cys= estimated GFR from CKD-Epi cystatin C equation, BIS_cr= estimated GFR from BIS-creatinine equation; BIS_cr-cys= estimated GFR from BIS creatinine-cystatin C equation, mGFR= measured GFR with iohexol.

Correctly Reclassified: CKD-EPI creatinine-cystatin C <60 and ^a^CKD-EPI creatinine (or ^f^CKD-Epi cystatin C, ^k^BIS creatinine, ^p^BIS creatinine-cystatin C) ≥60 ml/min/1.73m^2^. Incorrectly Reclassified: CKD-EPI creatinine-cystatin C ≥60 and ^b^CKD-EPI creatinine (or ^g^CKD-Epi cystatin C, ^i^BIS creatinine, ^q^BIS creatinine-cystatin C) <60 ml/min/1.73m^2^

Correctly Reclassified: CKD-EPI creatinine-cystatin C ≥60 and ^c^CKD-EPI creatinine (or ^h^CKD-Epi cystatin C, ^m^BIS creatinine, ^r^BIS creatinine-cystatin C KD cystatin C) <60 ml/min/1.73m^2^. ^d^Incorrectly Reclassified: CKD-EPI creatinine-cystatin C <60 and CKD-EPI creatinine (or ^i^CKD-Epi cystatin C, ^n^BIS creatinine, ^s^BIS creatinine-cystatin C KD cystatin C) ≥60 ml/min/1.73m^2^. NRI=net reclassification index; ^e^p-value=0.30, ^j^p-value=0.38, ^o^p-value=0.18, ^t^p-value=0.08.
