# Supplementary material for: Estimation of glomerular filtration rate from serum creatinine and cystatin C in octogenarians and nonagenarians
Source: BMC Nephrol. 2013 Dec 2;14:265. doi: 10.1186/1471-2369-14-265 (PMC4219437; doi:10.1186/1471-2369-14-265)
Supplement: Additional file 2: Table S2 — Reclassification of the participants with the use of the CKD-Epi creatinine-cystatin C equation versus: the CKD-Epi creatinine, the CKD-Epi cystatin C, the BIS creatinine or the BIS creatinine-cystatin C for estimated GFR, according to the cut-off value of mGFR = 45 ml/min/1.73 m2. [file 1471-2369-14-265-S2.doc]

Additional File 2, Table S2. Reclassification of the participants with the use of the CKD-Epi creatinine-cystatin C equation versus: the CKD-Epi creatinine, the CKD-Epi cystatin C, the BIS creatinine or the BIS creatinine-cystatin C for estimated GFR, according to the cut-off value of mGFR= 45 ml/min/1.73m^2^.

| Total group  N=95 | | Subgroup with mGFR <45 ml/min/1.73m^2^  N=26 | | | Subgroup with mGFR ≥45 ml/min/1.73m^2^  N=69 | | | NRI |
| --- | --- | --- | --- | --- | --- | --- | --- | --- |
| eGFR,  ml/min/1.73m^2^ | N (%)  Reclassified | Correctly Reclassified  N (%) | Incorrectly Reclassified  N (%) | Difference  (%) | Correctly Reclassified  N (%) | Incorrectly Reclassified  N (%) | Difference  (%) | (%) |
| CKD-Epi_cr | 17 (17.9) | ^a^  8 (30.8) | ^b^  0 (0) | 30.8 | ^c^  1 (1.4) | ^d^  8 (11.6) | -10.2 | 20.6^e^ |
| CKD-Epi_cys | 9 (9.5) | ^f^  2 (7.7) | ^g^  1 (3.8) | 3.9 | ^h^  5 (7.2) | ^i^  1 (1.4) | 5.8 | 9.7^j^ |
| BIS_cr | 10 (10.5) | ^k^  13 (11.5) | ^l^  0 (0) | 11.5 | ^m^  5 (7.2) | ^n^  2 (2.9) | 4.3 | 15.8^o^ |
| BIS_cr-cys | 2 (2.1) | ^p^  0 (0) | ^q^  0 (0) | 0 | ^r^  2 (2.9) | ^s^  0 (0) | 2.9 | 2.9^t^ |

eGFR= estimated GFR, CKD-Epi_cr= estimated GFR from CKD-Epi creatinine equation; CKD-Epi_cys= estimated GFR from CKD-Epi cystatin C equation, BIS_cr= estimated GFR from BIS-creatinine equation; BIS_cr-cys= estimated GFR from BIS creatinine-cystatin C equation, mGFR= measured GFR with iohexol.

Correctly Reclassified: CKD-EPI creatinine-cystatin C <45 and ^a^CKD-EPI creatinine (or ^f^CKD-Epi cystatin C, ^k^BIS creatinine, ^p^BIS creatinine-cystatin C) ≥45 ml/min/1.73m^2^. Incorrectly Reclassified: CKD-EPI creatinine-cystatin C ≥45 and ^b^CKD-EPI creatinine (or ^g^CKD-Epi cystatin C, ^i^BIS creatinine, ^q^BIS creatinine-cystatin C) <45 ml/min/1.73m^2^

Correctly Reclassified: CKD-EPI creatinine-cystatin C ≥45 and ^c^CKD-EPI creatinine (or ^h^CKD-Epi cystatin C, ^m^BIS creatinine, ^r^BIS creatinine-cystatin C KD cystatin C) <45 ml/min/1.73m^2^. ^d^Incorrectly Reclassified: CKD-EPI creatinine-cystatin C <45 and CKD-EPI creatinine (or ^i^CKD-Epi cystatin C, ^n^BIS creatinine, ^s^BIS creatinine-cystatin C KD cystatin C) ≥45 ml/min/1.73m^2^. NRI=net reclassification index; ^e^p-value=0.08, ^j^p-value=0.20, ^o^p-value=0.04, ^t^p-value=0.16.
